# Supplementary material for: Transcriptional landscape of soybean (Glycine max) embryonic axes during germination in the presence of paclobutrazol, a gibberellin biosynthesis inhibitor
Source: Sci Rep. 2019 Jul 3;9:9601. doi: 10.1038/s41598-019-45898-2 (PMC6610145; doi:10.1038/s41598-019-45898-2)
Supplement: Supplementary file 1 — Supplementary figures [file 41598_2019_45898_MOESM1_ESM.pdf]

Supplementary Material

Transcriptional landscape of soybean (*Glycine max*) embryonic axes during germination in the presence of paclobutrazol, a gibberellin biosynthesis inhibitor

Rajesh K. Gazara[1,#], Eduardo A. G. de Oliveira[1,#], Bruno C. Rodrigues[2], Rodrigo Nunes da Fonseca[2], Antônia Elenir A. Oliveira[1] and Thiago M. Venancio[1,*]

[1] Laboratório de Química e Função de Proteínas e Peptídeos, Centro de Biociências e Biotecnologia, Universidade Estadual do Norte Fluminense Darcy Ribeiro; Campos dos Goytacazes, Brazil. [2] Laboratório Integrado de Ciências Morfofuncionais, Núcleo em Ecologia e Desenvolvimento SócioAmbiental de Macaé (NUPEM), Macaé, Brazil. [#] These authors contributed equally to this work.

[*] Corresponding author
Av. Alberto Lamego 2000 / P5 / 217; Parque Califórnia
Campos dos Goytacazes, RJ
Brazil
CEP: 28013-602
thiago.venancio@gmail.com

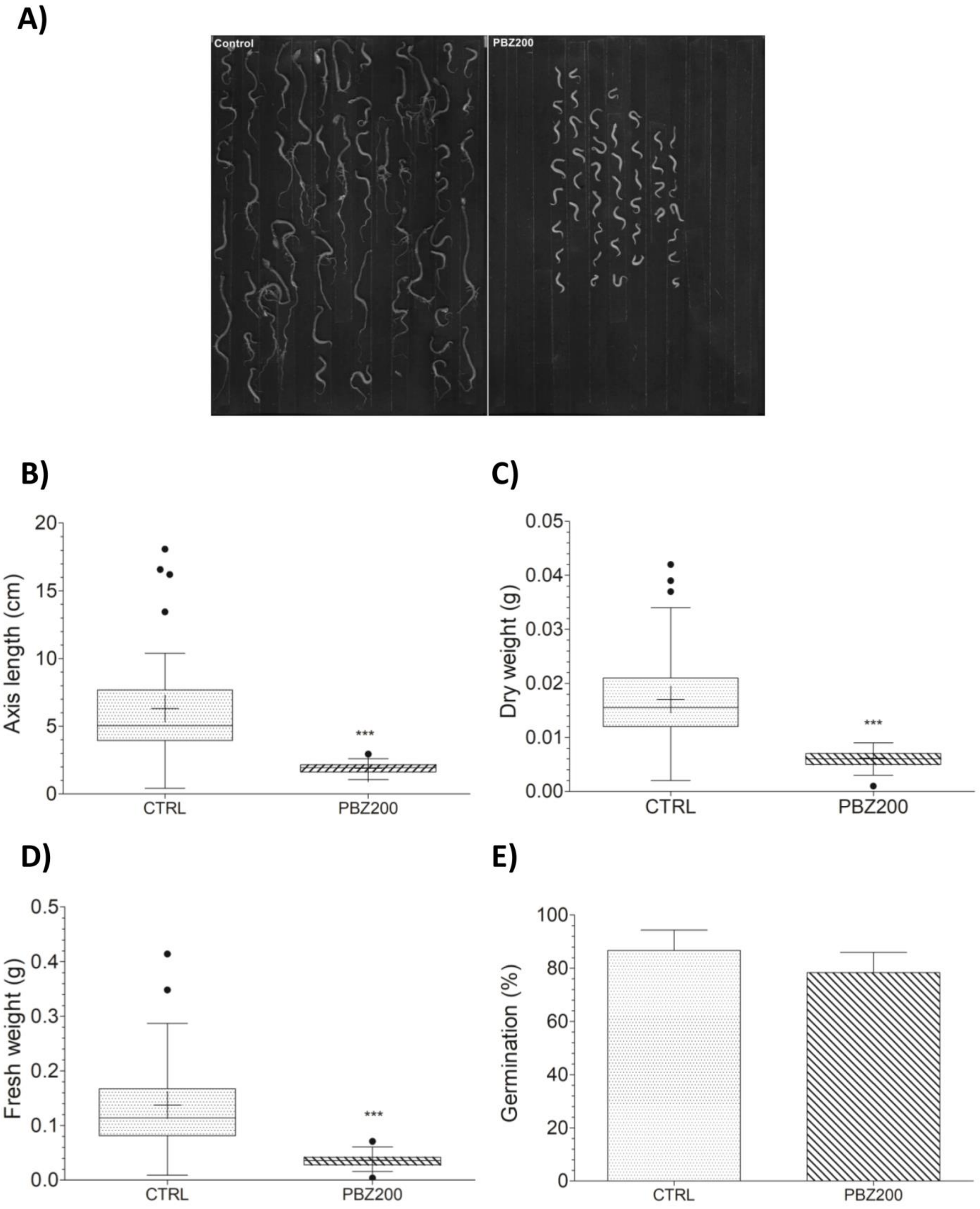

Supplementary figure S1. PBZ delays soybean seed germination. Soybean seeds (BRS-284) were allowed to germinate and grow in an incubation chamber under 28°C temperature, and 12/12h photoperiod (dark/light) for 7 days. Seeds were germinated in the presence of 30 ml of sterile water (control) or sterile water with 200 µM paclobutrazol (PBZ). **(A)** Photographs of embryonic axis submitted or not to PBZ treatment. **(B)** Embryonic axis length, **(C)** Dry weight, **(D)** Fresh weight, and **(E)** Seed germination. Asterisks above boxplot demonstrate significant difference ($p<0.0001$, Student´s T-test).

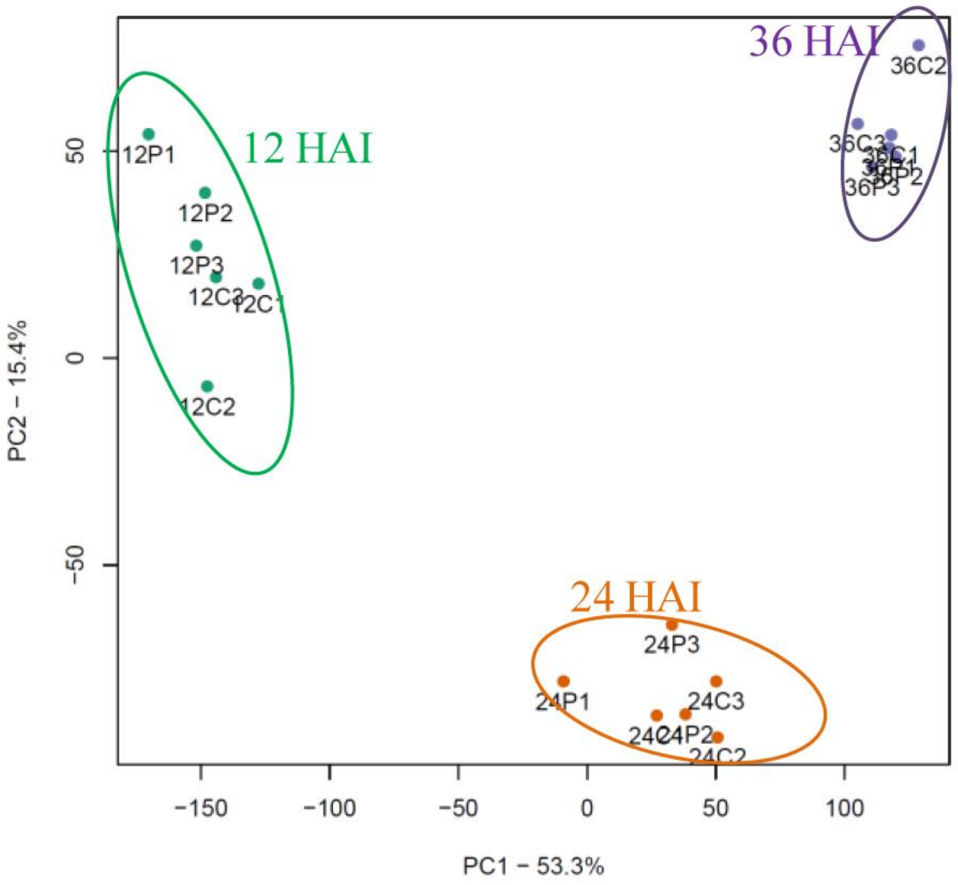

Supplementary figure S2. Principal Component Analysis (PCA) of expressed genes under control and PBZ at 12 HAI, 24 HAI and 36 HAI. The first PC (PC1) and second PC (PC2) explained 53.3% and 15.4% of the variances, respectively. Three distinct groups can be observed: 12 HAI (green), 24 HAI (orange) and 36 HAI (purple).

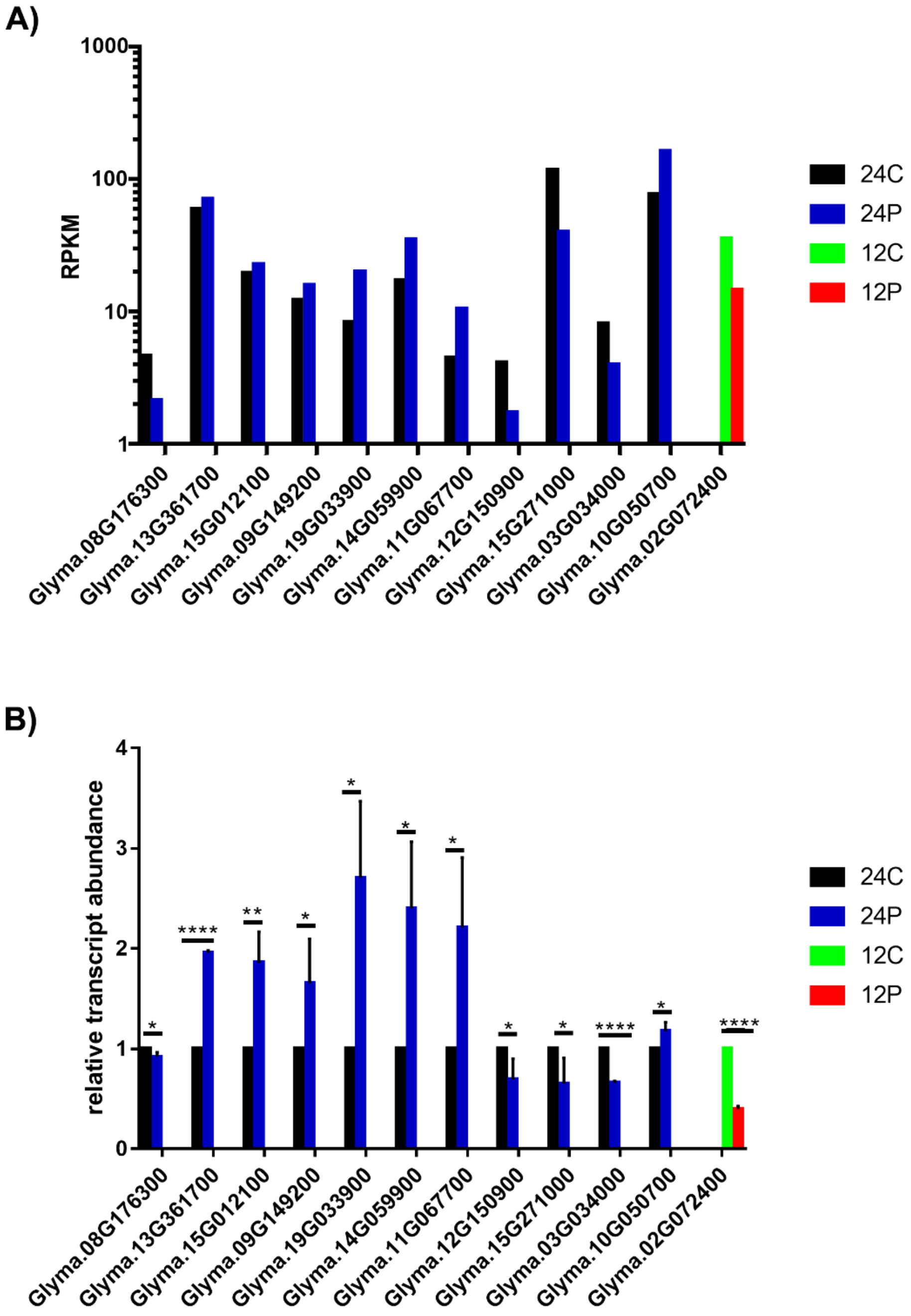

Supplementary figure S3. Validation of differential gene expression. **(A)** RNA-Seq expression levels of 12 genes selected for validation using Real-time qPCR. **(B)** Relative transcript abundance of selected genes, obtained by Real-time qPCR. X- and Y- axes represent gene identifiers and RPKM (panel A) or relative transcript abundance (panel B), respectively. Samples are represented in different colors, as follows: 24C, 24 hours after imbibition (HAI) without PBZ (control); 24P, 24 HAI, PBZ-treated; 12C, 12 HAI, control and; 12P, 12 HAI, PBZ-treated. Statistical significance levels were obtained by performing one-tailed T-tests for each gene, as follows: * ($p < 0.05$), ** ($p < 0.01$), and **** (0.0001).

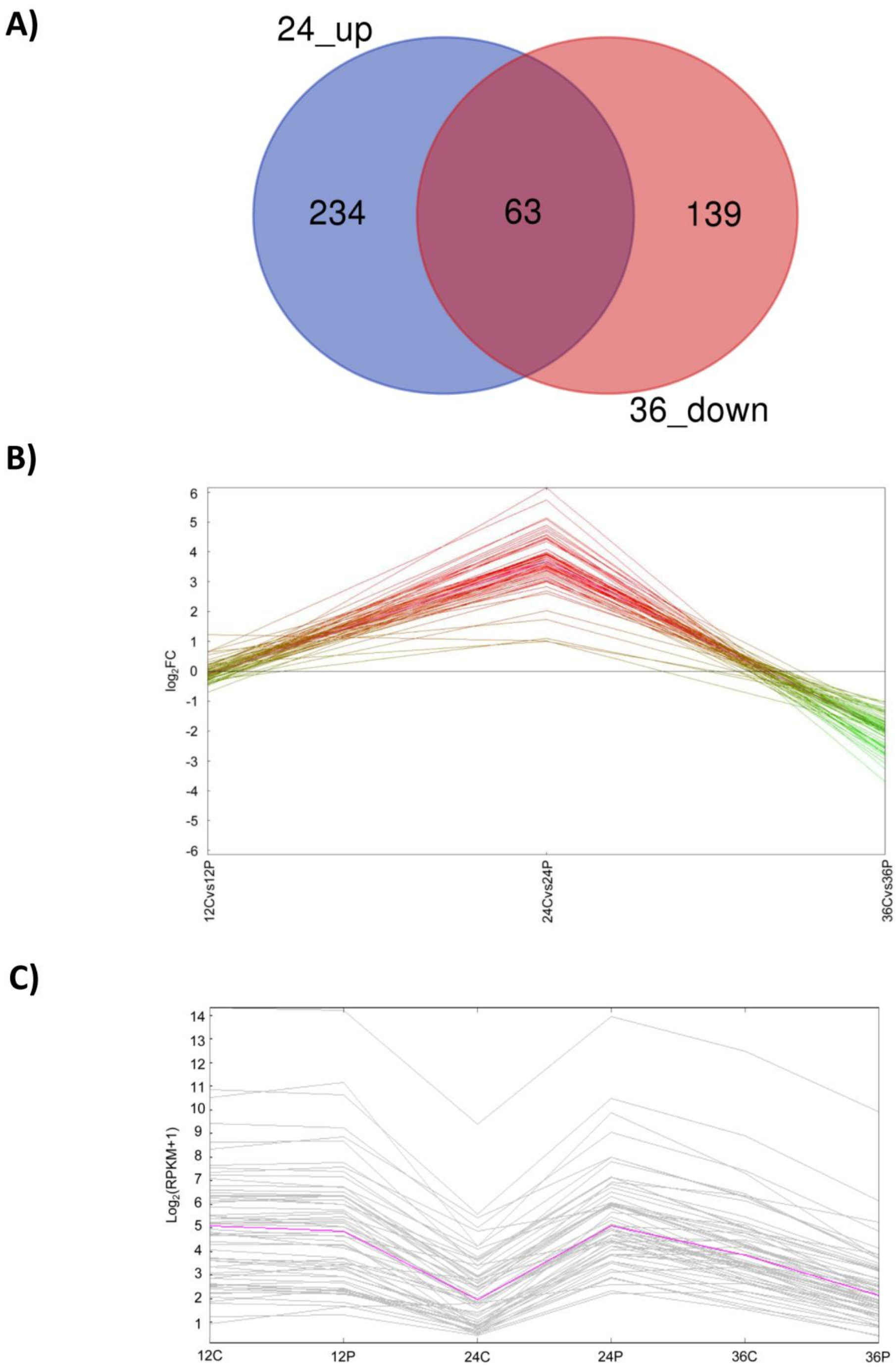

Supplementary figure S4. Overlap between 24-up- and 36-down-regulated genes. **(A)** Venn diagram (http://bioinformatics.psb.ugent.be/webtools/Venn/) shows 63 overlapping genes between 24 PBZ up- and 36 down-regulated genes. **(B)** Line plot of $\log_2$(fold change, FC) of the 63 genes at each time point. **(C)** Expression profile of the 63 genes in $\log_2$(RPKM+1) at each time point. MeV was used to generate graphs B and C. The pink line represents the median gene expression.
